# Is insulin-like growth factor-1 involved in Parkinson’s disease development?

**DOI:** 10.1186/s12967-020-02223-0

**Published:** 2020-02-11

**Authors:** Inma Castilla-Cortázar, Gabriel A. Aguirre, Giovana Femat-Roldán, Irene Martín-Estal, Luis Espinosa

**Affiliations:** 1 Tecnologico de Monterrey, Escuela de Medicina y Ciencias de la Salud, Ave. Morones Prieto 3000, 64710 Monterrey, N.L. Mexico; 2 Fundación de Investigación HM Hospitales, Madrid, Spain; 3 Centre for Tumour Biology, Barts Cancer Institute, Barts and The London School of Medicine and Dentistry, Queen Mary University of London, London, UK; 4 Neurocenter, Monterrey, Nuevo Leon Mexico

**Keywords:** Parkinson’s disease, IGF-1, Aging, Dopamine, Estrogens, Central nervous system

## Abstract

Parkinson’s disease (PD) is a neurodegenerative disorder that results in the death of dopaminergic neurons within the *substantia nigra pars compacta* and the reduction in dopaminergic control over striatal output neurons, leading to a movement disorder most commonly characterized by akinesia or bradykinesia, rigidity and tremor. Also, PD is less frequently depicted by sensory symptoms (pain and tingling), hyposmia, sleep alterations, depression and anxiety, and abnormal executive and working memory related functions. On the other hand, insulin-like growth factor 1 (IGF-1) is an endocrine, paracrine and autocrine hormone with several functions including tissue growth and development, insulin-like activity, proliferation, pro-survival, anti-aging, antioxidant and neuroprotection, among others. Herein this review tries to summarize all experimental and clinical data to understand the pathophysiology and development of PD, as well as its clear association with IGF-1, supported by several lines of evidence: (1) IGF-1 decreases with age, while aging is the major risk for PD establishment and development; (2) numerous basic and translational data have appointed direct protective and homeostasis IGF-1 roles in all brain cells; (3) estrogens seem to confer women strong protection to PD via IGF-1; and (4) clinical correlations in PD cohorts have confirmed elevated IGF-1 levels at the onset of the disease, suggesting an ongoing compensatory or “fight-to-injury” mechanism.

## Background: aspects and clinical features of Parkinson’s disease

Parkinson’s disease (PD) was first medically described as a neurological syndrome by James Parkinson in 1817. Over 50 years later, Jean Martin Charcot was more exhaustive in his descriptions and distinguished bradykinesia as a separate cardinal feature of this illness [1].

PD is the second most common neurodegenerative disorder, being age one of the main risk factors for its development [2, 3]. Despite years of study, much is still unknown about its etiology and pathogenesis, although genetic and environmental factors are involved in the establishment of the disease. However, the identification of such factors, their interaction and the associated molecular pathways that lead to neurodegeneration are weakly comprehended.

**Fig. 1:**
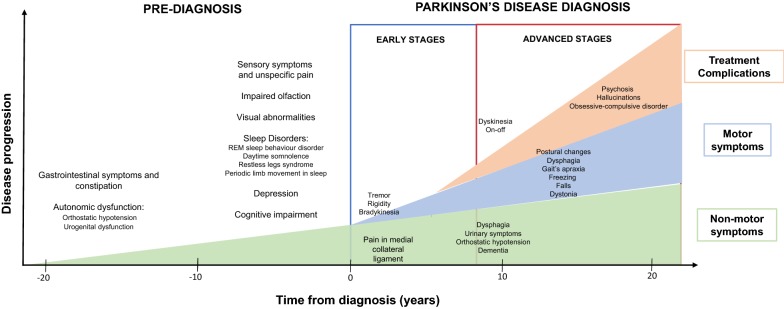
Chronology of clinical symptoms in Parkinson’s disease (modified from Kalia et al. [8]). Schematic representation of the diagnosis (even 10 to 20 years before the onset of the disease) and motor/non-motor symptoms in early and advanced stages of Parkinson’s disease, with clinical and other iatrogenic symptoms

There are multiple motor and non-motor symptoms that can develop in the course of PD (Fig. 1). This disorder is characterized by the appearance of progressive motor deficit, having four cardinal symptoms grouped in the acronym “TRAP”: tremor at rest, rigidity, bradykinesia and postural instability. To these, postural changes in flexion (camptocormia) and freezing are added as the most characteristic signs of the disease, impeding daily patient’s daily life activities [4, 5].

Tremor is the most common symptom of PD characterized by being at rest with unilateral and distal initiation at a frequency of 4–6 Hz, with a movement in prone-supine position like “counting coins”. This sign may occur in arms, legs, lips and jaw [5–8]. The rigidity observed in PD is described as an increase in muscle tone manifested by a resistance to passive movement. It is usually accompanied by a phenomenon called cogwheel [9]. Bradykinesia is depicted by difficulty in movement planning, initiation and execution [10]. Other manifestations associated with bradykinesia are the loss of spontaneous movements, hypomimia, hypophonia and deficit in arm-accompanying movement when walking [10, 11]. Postural instability appears late in the course of PD and is described as the loss of postural reflexes. These last alterations are frequent in PD patients, mainly camptocormia, which is characterized by neck, trunk, elbows and knees flexion. Nonetheless, other postural changes may occur, e.g. Pisa syndrome [10].

The freezing phenomenon is described as an inability to achieve movements, typically occurred during walking, producing severe disability. It usually lasts less than 10 s, but it can be prolonged. Freezing is accompanied by a festive march and it can be aggravated when turning around during the march or in closed spaces [9].

## Physiopathology of Parkinson’s disease

**Fig. 2:**
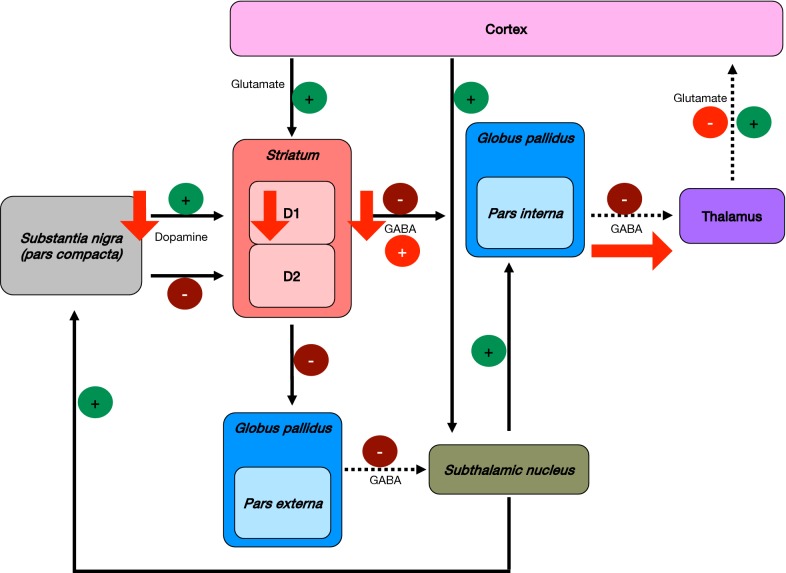
Basal ganglia dysfunction and physiopathology of Parkinson’s disease. The progressive loss of ascending dopaminergic projections is the key factor in the establishment of Parkinson’s disease. Continuous lines represent the normal function of basal ganglia; dotted lines represent the normal function of brain segments without the previous inhibition/activation pathway; and red arrows represent the alterations in basal ganglia observed in PD

The function of basal ganglia is determined by the balance between direct and indirect pathways activated by gamma-aminobutyric acid (GABA) neurotransmitter. The direct pathway starts with the activation of D1 dopamine receptors by dopamine in the *striatum*, which inhibit (via GABA) the *globus pallidus pars interna*. This last brain structure, in the absence of dopamine would inhibit the thalamus. Consequently, the direct pathway hinders the inhibition of the thalamus, thus stimulating the cortex (Fig. 2) [12]. On the other hand, the indirect pathway starts with the activation of dopamine D2 receptors in the *striatum*, inhibiting the *globus pallidus pars externa*, which in turn would inhibit (via GABA) the *subthalamic nucleus*. Due to the lack of inhibition by *globus pallidus*, the *subthalamic nucleus* activates the *globus pallidus pars interna*, which hinders (via GABA) the thalamus, hence inhibiting the cortex (Fig. 2) [12]. As follows, the direct pathway results in an excitation of the cortex, as opposed to the indirect pathway, where an inhibition of the cortex occurs.

The balance between these two pathways, regulated by dopaminergic afferent neurons in the *substantia nigra pars compacta* ascending to the *basal ganglia*, is progressively affected in PD. Thus, gradual loss of dopaminergic projections hinder the direct pathway, stimulating the *globus pallidus pars interna* that inhibit (via GABA) the thalamus leading to cortical dysfunction (Fig. 2) [12].

Motor activation alterations in PD patients reflect basal ganglia projection dysfunctions towards motor cortex as a result of the nigrostriatal degeneration, typified by progressive cell death in the central nervous system (CNS). The abnormal neuronal activity in areas receiving excessive inhibition of thalamo-cortical projections in PD has been evident in neuroimaging studies where they are hypoactive, such as the primary motor area (M1), supplementary motor area (AMA) and the lateral prefrontal cortex (DLPFC) [12].

Nonetheless, PD pathophysiology is not fully understood. It can be assumed that PD establishment depends upon various mechanisms, such as dopamine depletion, direct and indirect pathways alterations of the basal ganglia, dysfunction of the basal ganglia to the cortex and abnormal cortical plasticity [12]. PD motor manifestations begin focally, typically in one limb segment, when dopamine concentrations fall below 60–70% in the contralateral striatum [12].

**Fig. 3:**
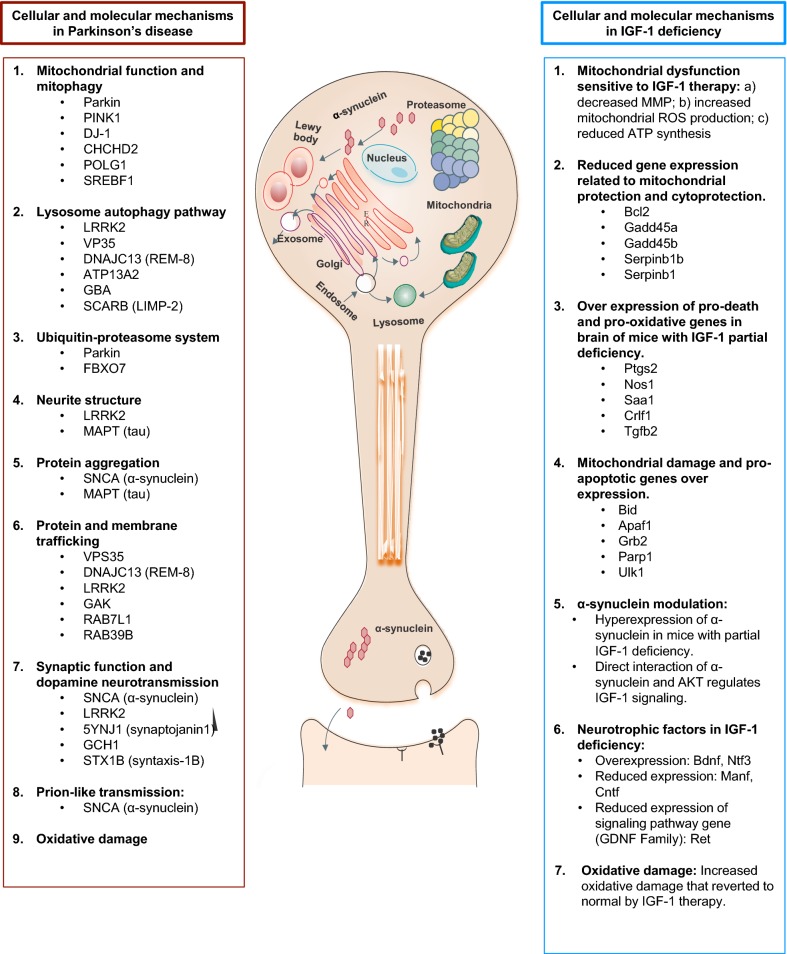
Parallelism between the cellular and molecular mechanisms involved in the pathogenesis of Parkinson’s disease and IGF-1 deficiency (modified from Kalia et al. [8])

The initial pathogenesis of PD consists in a cascade of events leading to cell death, which includes oxidative stress, excitotoxicity via glutamate pathways, impaired mitochondrial functions, protein misfolding and aggregation due to ubiquitin-proteasomal system dysfunction, altered lysosome and chaperone-mediated autophagy and the development of cytoplasmic inclusion bodies (called Lewy bodies) (Fig. 3, left panel) [13]. Inflammation and humoral immune reactions may contribute to cell death processes through apoptosis [12].

Lewy bodies contain neuro-filament proteins and ubiquitinated α-synuclein, being their presence an important factor that allows to classify pathological changes in PD progression: stage 1, involves changes in dorsal motor nucleus and olfactory bulb associated with the loss of olfactory function, commonly seen in pre-clinical PD; stage 2, includes Lewy body formation in *pons* and *medulla*; stages 3 and 4, produce clinical motor symptoms; and stages 5 and 6, involve damage in neocortical areas, that result in cognitive problems and dementia [14].

Oxidative stress is an alteration observed in PD and other neurodegenerative diseases, e.g. amyotrophic lateral sclerosis and Alzheimer’s disease. The brain is especially vulnerable to this oxidative environment, due to several factors: (1) the large fraction of oxygen consumed by this organ [15]; (2) its high metal ion levels that can react with specific reactive oxygen species (ROS) to form hydroxyl radicals responsible for the oxidative damage [16, 17]; and (3) the elevated prevalence of polyunsaturated fatty acids in brain membranes, which are particularly vulnerable to oxidative damage.

Recently, several studies in animal models have shown that various neurotrophic factors, when administered exogenously, reduce the vulnerability of dopaminergic neurons. One of these factors is the glial cell line-derived neurotrophic factor (GDNF), which seems to be effective in the treatment of PD [18].

As aforementioned, age is the major known risk factor for PD. It is important to notice that endogenous levels of some neurotrophic factors decrease with age. This idea suggests that neurotrophic factors loss due to aging may contribute to the etiology of PD [19].

## Prevalence of Parkinson’s disease

The prevalence of PD increases with age. To date, several meta-analyses have been conducted to obtain worldwide age and sex-related estimates for PD. In 2016, approximately 6.1 million people had PD, most belonging to high and middle socio-demographic index (SDI) countries, such as North America and Canada (Table 1) [3]. This rate is 2.4 times higher than in 1990 (2.5 million people with PD). However, such increase in PD patients worldwide between 1990 and 2016 is not explained entirely by the augmentation in elder people [3].

**Table 1:** Worldwide prevalence of Parkinson’s disease

Country	General population	References
High SDI countries	2.1 million (34.4%)	[3]
High-middle or middle SDI countries	3.1 million (50.8%)	
Low SDI countries	0.9 million (14.8%)	
Worldwide	6.1 million *Female*: 2.9 million (47.5%) *Male*: 3.2 million (52.5%)	

*SDI* socio-demographic index

Age-standardized prevalence of PD shows that this disorder is more frequent between 65 and 79 years, especially in men, being this ratio 1.40 times higher in men than women in 2016 (Table 2) [3, 20–27]. Consequently, additionally to elder age, male sex is recognized as a prominent risk factor in the development of PD. Also, PD incidence in men continues to rise after 80 years old; being the age of PD onset about 2 years later in women than men [28].

**Table 2:** Worldwide age-related prevalence of Parkinson’s disease

Age-related prevalence of Parkinson’s disease			
Age	Female	Male	References
4–49 years	3.26 per 10,000 person-years	3.57 per 10,000 person-years	Hirsch et al. [21]
> 80 years	103.48 per 10,000 person-years	258.47 per 10,000 person-years	

Age of Parkinson’s disease patients in countries with high prevalence of the disease			
Country	Age general population	References	
United States of America (USA)	70.5	Van Den Eeden [22]	
Japan	68.7 ± 10.3	Yamawaki et al. [26]	
Europe	61.6 ± 9.73	Pagano et al. [24]	
China	58.7 ± 10.55	Song et al. [25]	
Mexico	56.9 ± 11.8	Rodríguez-Violante et al. [23]	

## Parkinson’s disease, estrogens and sex differences. Estrogen actions via IGF-1 activity in brain

Estrogens are steroid hormones that promote female sex characteristics and reproductive capability in several organs, including brain. These hormones can cross the blood brain barrier and also the brain can endogenously produce estrogen from cholesterol [29].

Estrogens have been first identified for their role in female reproductive cycle. Nevertheless, their protective functions against chronic and degenerative diseases are becoming more relevant. These new roles may be responsible for the long-recognized advantages that women have over men in retaining their general health and achieving greater longevity [30].

Most of the circulating estrogens in premenopausal women are produced mainly by the ovaries, and in small quantities by other peripheral tissues, such as liver, adrenal gland, mammary glands, adipose tissue and brain [31]. In males, testes produce only 20% of circulating estrogens, being mostly locally produced by adipose tissue, brain, skin and bone. Herein, aromatases convert testosterone to estrogen [32].

Estrogen receptors have beneficial effects on memory and cognition, being widely distributed in male and female brains [33]. To date, several effects of estrogens in neurodegenerative diseases, such as PD, have been described [34]. Some of them are implicated in cognition, synaptic plasticity, memory, neurogenesis and neuroprotection [35]. Numerous studies have suggested that the molecular mechanism by which estradiol exerts its neuroprotective effects involves phosphatidyl inositol-3-kinase (PI3K) signaling pathway activation. This cascade is activated by insulin and insulin-like growth factor 1 (IGF-1) in brain [36]. As follows, there is a certain association between IGF-1 and estrogens and their protective effects exerted in nigrostriatal dopamine neurons after 6-hydroxdopamine lesions; suggesting them as an useful therapy to palliate PD symptoms [37].

In this manner, women with PD typically have a more benign phenotype with slower progression of the disease than men, and the incidence and prevalence of PD is higher in postmenopausal than in premenopausal women of similar age [38]. This could be due to the higher estrogen activity, which increases dopamine levels in the *striatum*, thus promoting neuron survival and neuroprotective actions [38, 39]. In addition, estrogens might prevent Lewy body deposition through specific α-synuclein anti-aggregation and fibril destabilization properties [40]. In the last decade, the modulation of α-synuclein aggregation is emerging as an innovative therapeutic strategy for PD treatment. In this fashion, small organic molecules, e.g. polyphenols such as curcumin, have been widely tested for their ability to inhibit α-synuclein aggregation [41, 42].

Until now, estrogen deficiency has been associated with PD. In industrialized countries approximately 0.3% of the entire population and near 1% of people older than 60 years have PD related to estrogen deficiency due to loss of function mutations [43]. For example, in European countries this prevalence ranges from 65.6/100,000 to 12,500/100,000 people, with an incidence from 5 to 346/100,000 [44–46].

Additionally, a range of behavioral and lifestyle preferences associated with gender differences, e.g. diet, exercise, smoking and caffeine, are emerging as potential PD risk modifiers [47]. The nigrostriatal dopaminergic pathway may thus underlie the biological sex differences vulnerability, and could also be responsible for the dimorphic sexual actions of estradiol, which shelter females against striatal dopamine loss in experimental PD. However, in males such pathway fails to protect or may even worsen striatal lesions. Studies in animal models with estrogen deprivation exhibit dopaminergic neuron loss, and altered dopaminergic metabolism and transporter uptake, which can be partially reverted by exogenous estrogen administration [48]. In consequence, there is a link between longer estrogen exposure during lifetime and both decreased PD risk and milder features in women [48]. All these results open a door to potential hormone-based therapies as novel approaches to develop treatments which can delay and possibly halt the progression of the disease [27].

## Physiological roles of insulin-like growth factor 1

IGF-1 is a 70-amino acid polypeptide hormone with several functions [49]. IGF-1 described roles include tissue growth and development, insulin-like activity, proliferation, pro-survival, anti-aging, antioxidant and neuroprotective, among others. Also, IGF-1 is a major relevant hormone in embryological and postnatal states [50]. Although it is mainly produced by the liver for endocrine activities, virtually every tissue is able to secrete IGF-1 for autocrine and/or paracrine purposes [51].

Until now, the best-known conditions of IGF-1 deficiency in humans are growth hormone insensitivity (GHI), advanced liver cirrhosis and aging, including cardiovascular and neurological diseases associated to aging [52]. More recently, intrauterine growth restriction [53] and metabolic syndrome [54] seem to be other forms of IGF-1 deficiency. Besides these conditions, several diseases have been recently proposed to be either consequence or cause of both systemic or local IGF-1 deficiency [52].

### Expression of IGF-1 in central nervous system

IGF-1 is produced by all major central nervous system (CNS) cell types, with an area-specific pattern production, being higher in the brain stem, cerebellum, cerebral cortex and the striatum [55, 56]. This hormone peaks during development, declining with age. IGF1 receptor (IGF1R) is expressed in both neural stem cells and all neural cells throughout life [57, 58], being highly produced among the choroid plexus, hippocampus and parahyppocampal areas [59]; but also to a less extent in the amygdala, cerebellum and cortex [60–62].

Several studies have shown that IGF-1 expression is increased in frontal cortex in PD compared to controls, while IGF1R is reduced in white matter and amygdala [63, 64]. As aforementioned, IGF-1 levels and signaling cascade are reduced during aging, due to an increased hyper-phosphorylation of insulin receptor substrates (IRSs), one of the first proteins activated in insulin and IGF-1 pathways, thus impairing the activation of downstream molecules such as PI3K [65–67].

IGF-1 mRNA expression levels in brain are relatively low compared to IGF1R, suggesting an important role for peripheral IGF-1 [68]. It was first reported in rats more than 20 years ago that systemic IGF-1 could cross the blood–brain-barrier by transcytosis [59] and via the choroid plexus [68]. Nonetheless, there is no significant correlation between serum and cerebrospinal fluid IGF-1 levels and therefore, it seems that systemic IGF-1 is not a major source of IGF-1 for the CNS [69]. For this reason, the endocrine role of serum IGF-1 is still a matter of debate.

### IGF-1 actions in the central nervous system

Little is known about the mechanisms that regulate IGF-1 expression in brain, but there is evidence that growth factors, nutrition, exercise and external insults (e.g. hypoxic/ischemic, demyelination, stereotactic, electrolyte and cryogenic injuries) influence IGF-1 expression in vivo [59, 70–74].

IGF-1 is a potent neuroendocrine regulator of the CNS development, maturation and plasticity [75], even being responsible for up to 40% of brain’s weight [76–80]. This hormone also has a role in brain differentiation, either from mesenchymal stem cells into neuronal lineages [81] or from neural stem cells towards neuronal or glial phenotypes [81]. IGF-1 has been found to exert profound effects on neurogenesis in the developing [78] and adult [82] brain, as well as in axonal development, synaptogenesis [68, 83] and migration [68, 84, 85]. Similarly, IGF-1 has shown to be indispensable for exercise-induced neurogenesis [86].

Several studies have found increased IGF-1 levels in the CNS following an injury, acting as an endogenous protective mechanism [87, 88]. This is in accordance with the increased IGF-1 levels observed at the beginning of PD pathophysiology (Table 3), suggesting that those whose IGF-1 concentrations do not rise enough will present a worst outcome. Similarly, murine models of peripheral nerve injury show that erythropoietin treatment promotes peripheral nerve regeneration by upregulating IGF-1 expression [89]. For these reasons, IGF-1 could be an important factor for brain protection and injury recovery, as it stimulates cell proliferation (progenitor, neuronal and non-neuronal) as well as their incorporation into the functional brain circuit [90], together with its anti-apoptotic, pro-survival and protective features on cellular metabolism [54].

**Table 3:** Worldwide Parkinson’s disease cohorts and IGF-1 levels

Patients	*n*	Age (SD)	Sex	Treatment	UPDRS-III stage (SD)	Design	Results	Peripheral blood IGF-1 (ng/ml) PD baseline/controls (SD)	References
Early (< 3.5 years) vs. moderate (> 4 years) PD	37	64 (7)	15F/22M	Levodopa	22 (9) (early)/38 (15) (moderate)	Longitudinal prospective cohort (3.5 years)^a^	PD patients in moderate, but not early stages, showed significantly increased baseline IGF-1 levels.	130 (26)/106 (24) *p *= *0.017*	Bernhard et al. [171]
Newly diagnosed idiopathic PD (Germany)	15	69 (8.3)	6F/9M	Drug-naïve	14.30 (5.3)	Cross-sectional cohort^a^	IGF-1 level was higher in patients with PD and inversely correlated with the UPDRS-III score (r = − 0.77) among PD patients IGFBP-3 unchanged IGF-1 level was not related to motor function in the healthy group	158.4 (40.4)/129.2 (29.1) *p *= *0.004*	Godau et al. [172]
Idiopathic PD (Germany)	18	67 (9)	8F/22M	Levodopa-treated vs. untreated	24.1 (8.5) (treated)/16.2 (4.1) (untreated)	Longitudinal (6 months)^a^	IGF-1 was significantly higher in treated PD patients than in controls at all time points (all *p *< *0.001*) IGF-1 levels were correlated with shorter disease duration (*r *= *0.56, p *< *0.001*) In the patient group, higher IGF-1 levels were correlated with shorter disease duration (*r1⁄4 0.56, p1⁄40.001*) In the healthy control group, higher IGF-1 levels were correlated with slightly impaired motor performance (*r1⁄40.46, p1⁄40.005*) In the untreated patient group, IGF-1 levels were significantly higher than healthy controls (*p *< *0.001*) Treatment did not alter GH	149.06 (30.3)/98.96 (23.2)	Godau et al. [173]
PD	38	68 (10)	F and M	–	–	Cross-sectional cohort^a^	Serum and CSF IGF-1 and IGFBP levels were higher in PD patients than controls (*p *< *0.001*)	CSF: 5.97 pg/mL (0.93)/4.40 pg/mL (0.58) Serum: 320.19 (40.86)/207.97 (19.51)	Mashayekhi et al. [174]
PD (75), multiple system atrophy (MSA, 25), and progressive supranuclear palsy (PSP, 16) (Japan)	116	68.1 (1.1)	44F/35M	Drug-naïve vs. levodopa-treated	26.9 (1.8)	Cross-sectional cohort^a^	Serum IGF-1 levels tended to be higher in early PD patients than controls There was a negative correlation between serum IGF-1 levels and age in PD patients and controls There was no significant correlation between disease duration and serum IGF-1 levels in PD patients In controls, there was no significant correlation between serum IGF-1 levels and UPDRS part III In PD and PSP patients, there was a negative correlation between serum IGF-1 levels and UPDRS part III. In early and drug naïve PD patients there was no significant correlation between serum IGF-1 levels and UPDRS part III IGF-1 serum levels in PD patients with HY stage 2 were significantly higher than those in PD patients with HY stages 3–5	130.3 (14.6)/114.4 (5.9)	Numao et al. [175]
> 3years PD with weight loss (11) vs. PD without weight loss (16)	27	63.5 (8.8)/60.5 (8.6)	6F, 5M/8F, 8M	Levodopa	43.45 (17.26)/37.75 (22.17)	Cross-sectional cohort^a^	BMI was lower in all PD patients Serum leptin levels were lower in all PD patients Serum GH and IGF-1 levels were higher in all PD patients, mostly in PD with weight loss and without weight loss, respectively Serum active ghrelin levels were positively correlated with serum IGF-1 levels in the control group (*p *< *0.05; r *= *0.67*) but not among PD patients	With weight loss 191.73 (33.84), without weight loss 152.19 (49.62)/144.17 (24.24) (*p *< *0.05* between PD patients and PD patients with weight loss vs. controls)	Fiszer et al. [176]
PD	25	67.9 (9.4)	5F/20M	Treated, drug not specified	–	Cross-sectional cohort^a^	IGF-1 and IGFBP-3 serum levels in PD patients showed no correlation with the duration and severity of the disease	132 (42)/113 (51)	Tuncel et al. [177]
Early PD (< 2 years) (Italy)	65	59.7 (8.3)	26F/39M	Drug-naïve	14.5 (6.7)	2-year follow-up prospective cohort^a^	At baseline, serum IGF-1 levels were significantly increased as compared to healthy controls A positive correlation between IGF-1 levels and a specific executive function (phonological fluency) assessing cognitive flexibility was found After a 2-year follow-up, IGF-1 levels were positively related to verbal episodic memory, visuoperceptual abilities and attention/executive functions Low IGF-1 levels at baseline were independently associated to poor performance on specific cognitive tasks assessing verbal episodic memory and executive functions after 2 years	91.6 (34.4)/79.1 (23) (*p *= *0.019*)	Pellecchia et al. [178]
Early PD (< 2 years) (Italy)	37	59.4 (9)	15F/22M	Drug-naïve	14.6 (7.1)	12-month follow-up prospective cohort^a^	At baseline, serum IGF-1 levels were moderately increased Patients at the highest IGF-1 quartile presented higher mean dopaminergic scores (worse outcome)	94.5 (37.5)/79.1 (23) (*p *< *0.011*)	Picillo et al. [179]
Early PD (< 2 years)	405	61.20 (9.7)	141F/264M	Drug-naïve	20.25 (8.93)	5-year follow-up prospective cohort^a^	IGF-1 levels were similar in PD and controls Lower serum IGF-1 levels were associated to poor performances in cognitive tasks assessing executive function, attention and verbal memory	136.6 (56.1)/134.45 (56.13)	Picillo et al. [180]
Meta-analysis covering de novo, drug-naïve idiopathic PD patients	166	–	–	Drug-naïve	–	–	Significantly higher serum IGF-1 levels among de novo, drug-naïve idiopathic PD patients at baseline		Li et al. [181]

*HY* Hoehn and Yahr score, *MDS-UPDRS-III* Movement Disease Society-modified UPDRS-III scale, *SD* standard deviation, *UPDRS-III* unified Parkinson’s disease rating scale

^a^Age, sex and body mass index (BMI, kg/m^2^), as well as the presence or absence of other medical factors known to affect IGF-1 levels, termed medical confounders: diabetes mellitus (reported in medical history or inferred by antidiabetic medication intake), beta-adrenergic medication, depression (and/or antidepressant medication), neuroleptic medication, thyroid dysfunction, inflammatory diseases and cancer. All of them were taken into account when the study was carried out

When unravelling neuronal plasticity-associated IGF-1 actions, the main mechanisms include modulation of glutamatergic receptor subunits, alterations in calcium channel conductance and potentiation of glutamatergic transmission (as a result of a reduction in GABAergic transmission) [82, 91–94]. Other mechanisms that have been proposed are the alteration in neural cell adhesion molecules and the stabilization of nascent blood vessels [95–97], in synergy with other growth factors, e.g. brain-derived growth factor (BDNF, where it enhances its activation) [98] or estradiol, as estrogen-induced plasticity seems to be IGF-1 interdependent [99, 100].

Moreover, the correlation found between aging, where IGF-1 levels decrease (known as somatopause) and cellular processes demonstrate that IGF-1 is a vital factor for brain homeostasis, due to its well-known activities as an energy loop regulator, cell protein sorter and cell communication [101]. What is more, within the brain areas that ensue life-long neurogenesis (e.g. the *dentate gyrus of the hippocampus*), a 60% reduction of neuron differentiation occurs during aging. This effect could be reversed by an intracerebroventricular injection of IGF-1 [62]. In addition to the aforementioned effects, more direct IGF-1 actions have been found in cognitive status [102, 103], amyloid clearance [70], resilience to insults [70] and behavior [104–106].

As it is known, astrocytes are the most abundant cell type in brain (the ratio astrocyte:neuron is 10:1), and the neuronal soma is entirely surrounded by astrocytic membranes [107]. For this reason, effects of a given molecule have to be determined, not only in neurons but in the entire web, particularly in astrocytes, in order to avoid overlooking results mediated through them. In this sense, astrocytes express IGF1R in the developing brain [107], and to a less extent in the adult [58], hence activating IGF-1 activities, such as proliferation [108], growth factor production [109, 110], cytoskeletal protein levels [108], intercellular communication and messengers [111–113], glutamate transport [114], glucose metabolism [115–117], and mitochondrial and membrane enzyme activity [118, 119].

The role of IGF-1 in adult astrocytes seems to be limited to modulate tissue damage response, where reactive astrocytes express high levels of IGF1R [120, 121] and IGF-1 [122]. Likewise, hepatocytes in damaged liver express IGF1R, a circumstance not observed under normal conditions [123–125]. In this context, pro-inflammatory signals activate astrocytes, turning them into activated pro-inflammatory cells that will secrete several factors (e.g. inducible nitric oxide synthase 2 -iNOS2-, cyclooxygenase 2 -Cox2-, tumor necrosis factor α -TNF-α-, etc.) contributing to neuron damage. IGF-1 anti-inflammatory capabilities transform these cells into anti-inflammatory ones, ceasing to produce pro-inflammatory signals and thus, enhance the production of neuroprotective molecules, such as humoral superoxide dismutase (SOD), neurotrophic factors, etc. Both anti- and pro-inflammatory cell types converge on the phosphatase calcineurin, that turns gene expression to one of this phenotypes [126]. Moreover, it has also been reported a neuroprotective role for IGF-1 associated to mitochondrial protection and antioxidant defenses in aging animals [127].

Data from our group suggest that the mere partial IGF-1 deficiency in mice (heterozygous mice, Igf1^+/−^, where one of the alleles of the Igf1 gene is disrupted) [128] increased brain oxidative damage, inflammation, edema, apoptosis and impaired learning and memory capabilities; being all these alterations restored by IGF-1 replacement therapy [129]. Consistently, these IGF-1 deficient mice have an alteration in the blood–brain barrier due to a distorted genetic expression of extracellular matrix and intercellular adhesion proteins (unpublished data). Furthermore, IGF-1 deficiency showed in this experimental model, an altered gene expression pattern encoding neurotrophic factors and their receptors [130].

While most of the results found in the literature have been discovered using in vitro studies, other were discovered using animal models of CNS injury or IGF-1 deficiency. Even though these experimental models have been really advantageous in unravelling the neurobiology of IGF-1, clinical data is very scarce and so is our understanding of its therapeutic potential. Nevertheless, tremendous possibilities have been appointed towards its usage. Herein we will focus on PD; however, the degenerative neurological conditions with the larger impact on population have been somehow linked with IGF-1 alterations [131–134].

## Neurodegenerative diseases related to aging as IGF-1 deficiency conditions

An increasing number of disorders have been reported highlighting the role of IGF-1 in many different organs and system functions. The knowledge concerning “IGF-1 deficiency conditions” or “GHI”, in which replacement therapy could be considered as an effective therapeutic strategy, is only recent.

The best-known condition of IGF-1 deficiency is Laron syndrome (also known as Laron dwarfism or GHI) [135, 136], predominantly found in children, characterized by alterations in growth hormone receptors (GHR) in the liver, and hence, reducing IGF-1 synthesis. In these patients, IGF-1 replacement therapy improves growth and development [137, 138].

Another IGF-1 deficiency condition, in this case in adults, is liver cirrhosis [139], where decreased IGF-1 serum levels were found in these patients. However, during the last decade, our research team has disclosed the causal link between IGF-1 deficiency and the severe malnutrition syndrome associated to liver cirrhosis progression. In fact, the exogenous administration of low doses of IGF-1 in experimental cirrhosis improved nutritional status and nitrogen balance [140], intestinal absorption [141–144], osteopenia [145, 146], testicular atrophy [147, 148], restored somatostatinergic tone controlling growth hormone (GH) secretion [149] and induced hepato-protective actions in the liver associated with a reduction in oxidative damage and enhancing mitochondrial protection [150–153]. In addition, IGF-1 replacement therapy increased albumin circulating levels in cirrhotic patients [154].

Thirdly, aging may be considered as a recognized IGF-1 deficiency condition [155–157]. Although several studies have suggested that reduced GH/IGF-1 activity promotes longevity [158–160], a significant amount of evidence indicates that IGF-1 might play a role in several pathological conditions [102, 161–165] commonly seen during aging, associated with oxidative tissue damage. Precisely, several reports suggest that age-related changes in cellular and tissue function are linked to a decrease in anabolic hormones, GH and IGF-1 levels [155–157, 166]. Accordingly, previous studies from our group reported that administration of low doses of IGF-1 in aging rats improved glucose and lipid metabolism, reducing insulin resistance, increased testosterone levels and serum total antioxidant capability of the cell, and reduced oxidative damage in brain and liver, all of them associated with a normalization of antioxidant enzyme activities and mitochondrial function [167, 168].

In addition to these well-known IGF-1 deficiency conditions and its correlation with aging and cognition, several neurodegenerative disorders show low IGF-1 serum levels, such as PD, Alzheimer’s disease and cerebrovascular disease [132, 134]. Conversely, several neurodegenerative diseases, with the exception of PD, exhibit reduced GH-stimulation test response (with clonidine, arginine and growth hormone stimulating hormone, GHRH) [169]. This result could partly explain why in PD, high serum levels of IGF-1 are found (Table 3), in opposition with its related diseases. Moreover, a study disclosed that 5-HT 1-receptor-mediated growth hormone secretion neurotransmission was absent in PD patients, suggesting an alteration in serotonin signaling [170].

As described before, the GH/IGF-1 axis is involved in brain development, growth and function; so its progressive decrease during aging could be embroiled in the establishment of several human cerebrovascular diseases, that lead to neuronal degeneration and dysfunction, such as PD, multiple system atrophy, Lewy body disease, Alzheimer’s disease, vascular dementia, amyotrophic lateral sclerosis, stroke, etc. [131–133].

## Theoretical relationship between Parkinson’s disease and IGF-1 deficiency

Noticeably, there is a clear relationship between IGF-1 and PD (Fig. 3). The preclinical data presented in this review strongly suggests a neuroprotective role for IGF-1. As Table 3 depicts, IGF-1 levels in almost all PD cohorts are higher in both sera and cerebrospinal fluid of these patients, particularly at the symptom’s onset (UPDRS-III) and then equals out as the disease progresses. These IGF-1 concentrations inversely correlate with the UPDRS-III score (i.e. with motor function), verbal episodic memory, visuoperceptual abilities and attention/executive functions; been not found such correlation on healthy subjects [171–181]. As it has been shown, lower IGF-1 levels at the beginning of PD are associated with a worse disease progression and prognosis. However, IGF-1 correlation with the disease onset, duration, severity, etc., still remains a hot topic.

Few genetic alterations have been linked to PD, being the most common the so-called idiopathic PD. Among them are mutations affecting ɑ-synuclein [182–184], which leads to familial PD. However, the exact role of ɑ-synuclein in the pathophysiology of PD, besides its aggregation to form Lewy bodies, remains unclear [185]. Recently, ɑ-synuclein has been found to be essential for IGF-1 and other neurotrophic factors signaling activation in brain via protein kinase B (PKB or Akt). Actually, it has been hypothesized that ɑ-synuclein long-unknown role in brain could be to transport, localize and solubilize Akt for neurotrophic factor receptors in brain cells [186]. In vitro studies have disclosed that decreased Akt phosphorylation due to lack of functional ɑ-synuclein lead to poor IGF-1 signaling in neuronal and non-neuronal cells. This molecule, Akt, is a key survival signaling mediator of IGF1R in all cells, suggesting that IGF-1 protective effects in brain could be halted at the PD etiology itself.

Also, it has been proved that ɑ-synuclein aggregation neurotoxicity is mediated by dopamine, and that IGF-1 confers protection against such aggregation and promotes signaling rescue through Akt, even in presence of dopamine [187]. Of interest, a recent study found an accurate micro-RNA profile alteration in post-mortem microdissected dopaminergic neurons. In this study, miR-126 particularly blocked IGF-1 signaling pathway, increasing cell vulnerability to 6-hydroxydopamine neurotoxicity [188]. In consequence, all this data demonstrates that cell response capability is decreased may be due to ɑ-synuclein, miR-126, or dopamine cytotoxicity, dysregulating IGF-1 signaling pathway that lead to cell death and neurodegenerative features observed during the course of PD.

Several epidemiological studies have demonstrated a significantly increased risk for developing PD in individuals with central obesity [189, 190], an important result because IGF-1 and insulin signaling pathways are known to be dysregulated under metabolic derangements [54]. This suggests that insulin/IGF-1 signaling deficit could decrease neuroprotection and render the brain vulnerable to oxidative damage [191]. Furthermore, epidemiological studies have shown an association between the exposure to environmental toxins and an increasing incidence of PD in younger people [192–194]. Modern era exposure to toxins causes an elevated chronic grade of inflammation, alike obesity, that in turn antagonizes insulin/IGF-1 signaling, generating resistance and rendering the body without its physiological, trophic and protective effects. This assertion has been recently intimately linked to cardiovascular diseases [195].

As aforementioned, results from PD cohorts illustrated in Table 3 shows that lower IGF-1 levels at the beginning of the disease are associated with a worse progression and prognosis, being IGF-1 concentrations higher in PD cohorts at the symptom’s onset. These data could act as a compensatory mechanism due to the lack of IGF-1 signaling feedback and, also, it could be an endogenous protective factor released in response to cell death and oxidative damage promoted by the disease itself. Such hypothesis is further sustained by a progressive PD model study where IGF-1 was found to protect the nigrostriatal pathway, and that this protection was preceded by activations of key pro-survival signaling cascades, such as Akt [181, 196].

## Conclusions

There are strong lines of evidence to correlate IGF-1 with PD establishment and development. Herein, the present review tries to summarize how IGF-1 and estrogen levels, together with their neuroprotective and anti α-synuclein aggregation properties, act in a harmonized manner to maintain brain homeostasis, particularly during aging process. From our experience, the mere partial IGF-1 deficiency in a murine model increases brain oxidative damage, inflammation, edema and apoptosis, also impairing learning and memory capabilities and the blood–brain barrier integrity. All these alterations are restored by exogenous IGF-1 replacement therapy.

As it is known, the pathogenesis of PD, the second most common neurodegenerative disorder, consists in a cascade of events including oxidative stress, excitotoxicity via glutamate pathways, impaired mitochondrial functions, protein misfolding and aggregation due to ubiquitin-proteasomal system dysfunction, altered lysosome and chaperone-mediated autophagy and the development of cytoplasmic Lewy bodies, eventually leading to cell death. Due to its neuroprotective functions, in the last decade several studies have been conducted in order to understand and resolve the linkage between IGF-1 and PD. In this way, numerous basic and translational reports have appointed brain protection and injury recovery functions of IGF-1 in CNS injuries and/or PD patients. These results highlight the protective and homeostatic key roles of IGF-1 in brain, along with its anti-apoptotic, pro-survival and protective features on cellular metabolism.

The aging process, where IGF-1 serum levels decrease with age, is one of the risk factors for PD development. However, several clinical correlations in PD patients have reported an augmentation in IGF-1 levels at the onset of the disease, which suggests, given the preclinical results, an ongoing compensatory or “fight-to-injury” mechanism, since as the disease progresses such elevation dissipates. Additionally, lower IGF-1 levels at the beginning of PD are associated with a worse disease progression and prognosis.

Together with age, male sex is another crucial risk factor for PD establishment. Estrogens seem to confer women a strong protection to PD due to their beneficial effects on memory and cognition. This estrogen related neuroprotection is achieved via IGF-1 signaling pathway, as estrogens activate PI3K to stimulate neuron survival and protection. Nevertheless, more experimental studies need to be performed to better understand the molecular mechanisms from IGF-1 cascade involved in PD.

In this fashion, it could be reasonable to think in a combined therapy with estrogens and IGF-1, since both molecules can cross the blood–brain barrier activating the IGF-1 signaling pathway to exert its favorable actions in brain and also to prevent α-synuclein aggregation (Lewy body deposition), thus avoiding PD development. This hypothesis is in accordance with recent studies where polyphenols, such as curcumin, are used to inhibit α-synuclein aggregation and prevent oxidative damage related alterations. Also, GDNF, a neurotrophic factor, could be used as a therapy for PD, because it can reduce the vulnerability of domapinergic neurons. However, in order to solve this question, more cexperimental and clinical studies need to be done.

## Data Availability

Not applicable.

## Abbreviations

BDNF: brain-derived growth factor
CNS: central nervous system
Cox2: cyclooxygenase 2
GABA: gamma-aminobutyric acid
GDNF: glial cell line-derived neurotrophic factor
GH: growth hormone
GHI: growth hormone insensitivity
GHR: growth hormone receptor
GHRH: growth hormone releasing hormone
IGF-1: insulin-like growth factor 1
IGF1R: IGF-1 receptor
iNOS2: inducible nitric oxide synthase 2
IRSs: insulin receptor substrates
PD: Parkinson’s disease
PI3K: phosphatidyl inositol-3-kinase
PKB: protein kinase B
ROS: reactive oxygen species
SDI: socio-demographic index
SOD: superoxide dismutase
TNF-α: tumor necrosis factor α
